# Consultant psychiatrist/MPAC roles and the importance of specialist expertise

**DOI:** 10.1192/bjb.2026.10229

**Published:** 2026-06

**Authors:** Celina Sharma, Baxi Sinha

**Affiliations:** LD Psychiatry, Cumbria, Northumberland, Tyne and Wear NHS Foundation Trust, Gosforth, UK; Stockton Affective Disorders Team, Tees, Esk and Wear Valleys NHS Foundation Trust, Stockton-on-Tees, UK

The recent job advertisement for a Consultant Forensic Psychiatrist/Practitioner MPAC at Somerset NHS Foundation Trust (Ash Ward),^1^ closing December 2025, reflects a shift towards combination of consultant duties with the Multi-Professional Approved Clinician (MPAC) role. Although MPACs can contribute valuable perspectives, strengthen multidisciplinary team functioning and share workload, preservation of the specialised knowledge and skills, leadership role, and statutory accountability of consultant psychiatrists is crucially important. The extensive training of a consultant psychiatrist, which includes intense higher training, MRCPsych examination, and years of supervised clinical practice, ensures that they are capable of managing complex case presentations, providing leadership, and upholding safe and effective care under the Mental Health Act.^2^

In a staff survey conducted in Tees, Esk and Wear Valleys NHS Foundation Trust, completed by 60 of 90 consultants and SAS doctors, although respondents appreciated the contributions of MPACs, a significant number of doctors expressed concerns regarding role clarity, compromise of medical expertise, and increased reliance on and expanded responsibilities for medical colleagues.^3^ Respondents consistently emphasised that MPACs function best when complementing rather than substituting for a consultant psychiatrist. This highlights the importance of maintaining the unique roles of medical professionals.

The growing use of consultant/MPAC job descriptions may give the impression that consultant psychiatrists can be substituted by other professionals. Consultant psychiatrists hold a level of medical responsibility that cannot be transferred without affecting patient safety. They are central to providing stability within a service, supporting other resident doctors of all levels, supervising approved clinicians from other professions and ensuring continuity of care. Trainees are also affected; although psychiatric trainees recognise the value added by the multi-professional workforce, such advertisements may leave them questioning how their medical expertise is distinguished and valued within services, resulting in a huge impact on their morale and career motivation.

Rather than merging the roles, services should clearly emphasise the complementary contributions of both consultant psychiatrists and MPACs. Whereas MPACs provide valuable perspectives that strengthen multidisciplinary teamwork, consultants deliver indispensable clinical leadership, oversee risk management, maintain statutory responsibilities, and provide teaching and training to resident doctors. Both positions are crucial for effective operation of secondary mental health services. Yet they are not interchangeable. Consequently, when NHS trusts define these roles clearly and respectfully in job descriptions, they help to ensure a high quality of care, as well as strengthening staff morale and fostering an environment that benefits both patients and clinicians.
